# Repeatability and reproducibility of MRI-derived radiomics on a 0.35 T MR-Linac using a tissue-mimicking phantom

**DOI:** 10.1016/j.phro.2026.101035

**Published:** 2026-07-07

**Authors:** Florian Collard, Fanny Herault, Ludovic Vanquin, Anne-Laure Gagez, Alain Loussert, Dominique Collard, David Pasquier

**Affiliations:** a E-Health Department, ISEN-Méditerranée, Toulon, France; b Academic Department of Radiation Oncology, Centre Oscar Lambret, Lille, France; c Medical Physics, Centre Oscar Lambret, Lille, France; d Department of Clinical Research and Innovation, Centre Oscar-Lambret, Lille, France; e IM2NP UMR 7334, CNRS, Aix-Marseille Université, Marseille, France; f Institute of Industrial Science, The University of Tokyo, Tokyo, Japan; g Lille Univ., CRIStAL UMR 9189, Lille, France

**Keywords:** MR-Linac, Radiomics, Quantitative imaging, Magnetic resonance imaging, Feature reproducibility, Phantom study, Preprocessing pipelines

## Abstract

**Background and purpose:**

Magnetic resonance–guided radiotherapy provides real-time soft-tissue–based targeting during dose delivery. Beyond image guidance, magnetic resonance linear accelerator (MR-Linac) systems offer new opportunities for developing magnetic resonance imaging (MRI)-derived radiomics biomarkers. However, the reproducibility of these biomarkers in a low-field MR-Linac environment remains unclear.

**Materials and methods:**

This study evaluated the repeatability and reproducibility of MRI-derived radiomic features acquired on a 0.35 T MR-Linac using a tissue-mimicking phantom containing multiple tissue-equivalent materials. Fifty balanced steady-state free precession acquisitions were performed over 10 sessions, enabling both intra- and inter-session analyses. Forty-eight preprocessing pipelines combining normalization, discretization, and bias-field correction were assessed using the coefficient of variation and intraclass correlation coefficient for 68 radiomic features across 12 defined regions of interest.

**Results:**

The combination of N4 bias correction and z-score normalization yielded the highest overall stability, with several first-order (e.g. Entropy, Mean) and texture-based (e.g. glcm_DifferenceEntropy and glrlm_ShortRunEmphasis) features showing a coefficient of variation <5% and/or an intraclass correlation coefficient (2,1) > 0.85 across phantom materials. Stability varied with phantom composition, with higher reproducibility in homogeneous PVP-40 and water inserts compared to fat or fibroglandular compartments. Comparison with previous 0.35 T MR-Linac studies identified a consistent subset of robust features, supporting their potential as standardized MRI-derived radiomic biomarkers.

**Conclusions:**

These findings demonstrated the critical role of preprocessing and tissue composition in feature reproducibility and highlighted the importance of tissue-mimicking phantoms for validating MR-Linac-based quantitative imaging pipelines.

## Highlights

• A 0.35 T MR-Linac acquired 50 SSFP scans of a 4-material phantom.
• Sixty-eight IBSI-compliant radiomic features were extracted from 12 ROIs.
• Forty-eight preprocessing pipelines were tested across 10 imaging sessions.
• Stability used CoV ≤5%, ICC(2,1) > 0.85, and FDR-Wilcoxon testing.
• N4 + z-score/128–512 scaling yielded the best CoV and ICC(2,1) stability.

## Graphical abstract

**Figure:**
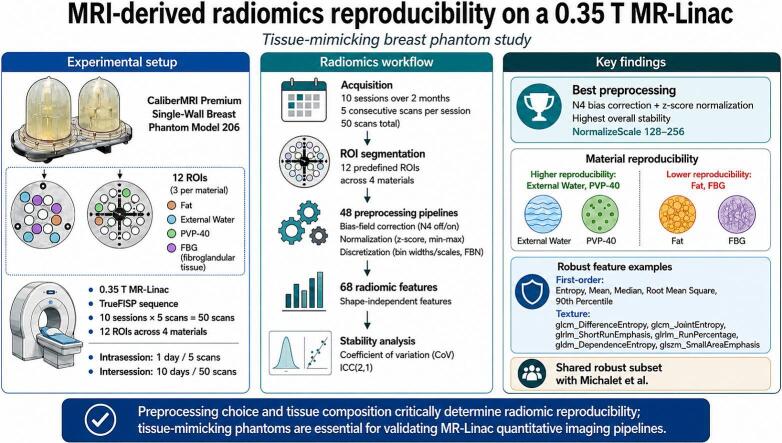

## 1 Introduction

Magnetic resonance (MR)-guided radiotherapy (MRgRT) is a major technological development in modern radiation oncology. By integrating magnetic resonance imaging with a linear accelerator (Linac), MR-Linac platforms enable precise dose delivery with high-contrast, non-ionizing, real-time imaging [1]. This hybrid configuration allows continuous visualization of anatomical structures during treatment, facilitating accurate target localization, adaptive planning, and improved sparing of healthy tissues. MR-Linac systems are being explored in breast radiotherapy, where improved soft-tissue contrast may enhance visualization of the tumor bed and surrounding glandular tissue, potentially supporting margin reduction and adaptation to inter-fraction anatomical changes.

Beyond image-guided radiotherapy, MR-Linac systems provide opportunities for quantitative imaging, including radiomics and parametric magnetic resonance imaging (MRI) approaches, with the potential to derive imaging biomarkers that predict or monitor tumor response to treatments. The AAPM 294 report [2] identifies promising avenues for personalizing MRI biomarkers and improving clinical outcomes. However, no MRI-derived biomarker has been validated for routine clinical use, and MR-Linac studies of radiomic feature performance and quantitative MRI remain limited, despite recent work [3]. Michalet et al. [4] investigated radiomic feature reproducibility on a 0.35 T MR-Linac using phantom and patient data, showing that many features lack reproducibility. Reliable quantitative imaging requires repeatable and reproducible quantitative metrics [5], with robustness and standardization assessed across acquisition, reconstruction, segmentation, and feature extraction [6]. Radiomics provides a useful framework as it extracts quantitative features from medical images that may serve as prognostic or predictive biomarkers in radiotherapy [7], [8], [9].

Phantom-based experiments are recommended to provide controlled, reproducible conditions [10]. Tissue-mimicking phantoms allow quantitative imaging performance to be assessed across materials representing different biological tissues, providing a realistic reference for benchmarking and optimizing imaging pipelines.

This study assessed the repeatability and reproducibility of MRI-derived radiomic features from a 0.35 T MR-Linac by evaluating multiple image-processing pipelines on a tissue-mimicking phantom. Building on MR-Linac reproducibility studies [11], including phantom-based analyses such as Michalet et al. [4], this work used a multi-material tissue-mimicking phantom for material-specific reproducibility analysis and systematic evaluation of preprocessing pipelines.

## 2 Materials and methods

**Fig. 1:**
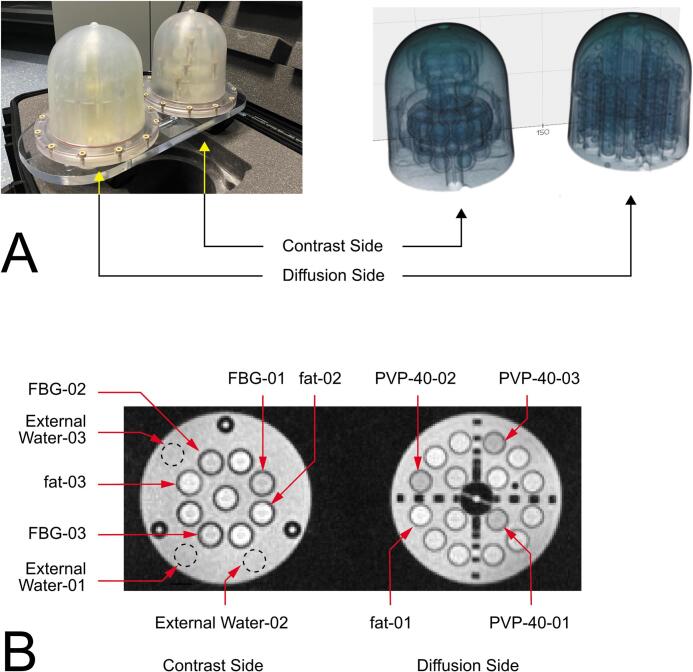
Overview of the phantom and material mask definition. A left: Photograph of the CaliberMRI phantom showing its two compartments—The contrast side and the diffusion side. Right: Example of MRI-based segmentation delineating the various material inserts embedded within the phantom B: Definition and spatial localization of material-specific masks used for radiomic feature extraction. The coronal slice was positioned 120 mm from the phantom reference plane (1), corresponding to a section near the base

Image preprocessing pipelines were evaluated by their ability to preserve the consistency of radiomic features extracted from repeated MRI acquisitions of the CaliberMRI Premium Single-Wall Breast Phantom Model 206 [12] (Fig. 1). Preprocessing was applied uniformly across all images, and features were computed in non-overlapping phantom regions, each containing a unique material. Feature repeatability and reproducibility under each preprocessing strategy were assessed separately for each material using the coefficient of variation (CoV) and intraclass correlation coefficient (ICC(2,1)).

### 2.1 Phantom

The Premium Single-Wall Breast Phantom Model 206 (CaliberMRI) [12] is a high-precision MRI phantom developed for quantitative breast and soft-tissue imaging. This study used imaging data from the contrast and diffusion compartments, containing fibroglandular (FBG) inserts, fat mimics, high-performance liquid chromatography-grade water, and polyvinylpyrrolidone (PVP) solutions at varying concentrations to simulate a range of tissue diffusion properties.

### 2.2 Imaging

All scans were performed using a 0.35 T MR-Linac system (MRIdian®, ViewRay Inc., Oakwood Village, OH, USA) with a balanced steady-state free precession TrueFISP sequence, routinely used in MR-Linac systems for real-time imaging and treatment guidance because of its high signal-to-noise ratio and rapid acquisition. The resulting images comprised a matrix of 168 × 168 × 268 voxels, with isotropic in-plane resolution and a voxel size of 1.5 × 1.5 × 1.5 mm^3^. Acquisition parameters included a field of view (FOV) of 40 × 25 × 25 cm^3^ and an average acquisition time of 1 min 7 s.

Ten imaging sessions were conducted over 2 months. In each session, five consecutive acquisitions were performed without phantom repositioning. This design enabled evaluation of two temporal hierarchies: (i) intrasession repeatability, assessed from five consecutive acquisitions within each session, and (ii) intersession reproducibility, assessed across all 50 scans. Between sessions, the phantom was repositioned using a laser-assisted alignment procedure with positioning accuracy exceeding the smallest voxel dimension (see Alignment Technique for details). Before each acquisition, the phantom temperature was stabilized and maintained at 22 ± 1 °C using the integrated MR-readable, liquid crystal (LC) thermometer to ensure consistent imaging conditions.

### 2.3 Masks

Twelve masks were generated within the phantom voxel matrix, with three masks for each of the four insert types listed in Table 1 and shown in Fig. 1. Selected inserts represented intensity profiles and textures simulating different anatomical and cellular compositions. Segmentation used an interactive Python tool incorporating SimpleITK and NumPy and based on region-growing from a user-selected coronal seed voxel. A voxel was included when its intensity was ≥ seed_value −28 (untreated full-image range ≈800 gray levels) and at least three adjacent faces were already included. A 3D morphological erosion with a cross-shaped 1-voxel-radius kernel cleaned borders and prevented leakage into irrelevant regions.

**Table 1:** Definition of the insert materials contained in the masks.

Insert type	Mimic material	Abbreviation	Intended contrast
Fat	Fat-equivalent oil	fat	Low signal, lipid-rich composition
External Water	HPLC water	ext	High, homogeneous signal
PVP-40	40% poly-vinyl-pyrrolidone	pvp-40	Intermediate, homogeneous signal
FBG (Fibroglandular)	Dense-tissue mimic	fbg	Heterogeneous signal

HPLC, High-Performance Liquid Chromatography grade.

If region-growing did not converge, a standardized cylindrical mask was automatically placed at the position maximizing voxels within ±28 units of the seed. Final masks were saved in NIfTI (.nii) format to preserve DICOM geometry. Segmentation was performed once on a reference image; masks were then applied identically to all subsequent acquisitions after voxel-level visual verification and software-based checks confirmed spatial alignment and consistency. Supplementary Fig. S1 shows representative mask overlays.

### 2.4 Software architecture

The workflow was managed by a main control script that detected DICOM-series folders, linked them to corresponding NIfTI masks, and executed the pipeline from preprocessing to radiomic feature extraction.

Processing functions were organized in a dedicated Python library (irmtech_lib.py) including optional N4ITK intensity bias correction, normalization methods (min-max, z-score, and fixed bin number discretization [FBN]), and caching mechanisms for repeated computations.

### 2.5 Preprocessing

A total of 48 unique configurations were evaluated by combining four preprocessing strategies with and without N4 bias field correction. All operations were applied identically across image volumes following IBSI guidelines [13].

The pre-processing methods were:

• N4 Bias Field Correction (optional): Correction of low-frequency intensity non uniformities using the N4ITK algorithm [14].
• Fixed bin-width discretization (BinWidth): Intensity discretization using fixed bin widths of 1, 2, 5, 10, 20, and 50.
• z-score Normalization + scaling (z-score + NormalizeScale): Standardization of voxel intensities to zero mean and unit variance, followed by linear scaling to discrete intensity levels of 8–512 (BinWidth = 1).
• Min-Max Normalization + discretization (MinMax + BinWidth): Scaling to the 0–65,535 range, followed by bin widths of 64–1024.
• FBN: Discretization into 32–1024 fixed bin counts.

### 2.6 Radiomic feature extraction

Radiomic features were extracted from normalized NIfTI images using PyRadiomics (v3.1.0) [15], with regions of interest defined by the 12 binary masks above. Each mask was analyzed independently within its corresponding compartment. No spatial filtering, such as wavelet or Laplacian of Gaussian, was applied. Sixty-eight radiomic features were extracted, including first-order statistics, Gray-level co-occurrence matrix (GLCM), Gray-level run length matrix (GLRLM), Gray-level-size zone matrix (GLSZM), neighboring gray-tone difference matrix, and Gray-level Dependence Matrix (GLDM) features. Shape features and other volume-dependent metrics, including Energy, Total Energy, Mesh Volume, Surface Area, Compactness, and Sphericity, were excluded. Results were compiled in a centralized CSV file with metadata describing preprocessing strategy and mask identity, ensuring traceability and reproducibility.

### 2.7 Statistical analysis

Feature stability was evaluated using CoV and ICC(2,1) across repeated acquisitions with automated Python scripts. For each mask group, i.e., three masks corresponding to the same insert type in Table 1 and Fig. 1, CoV was computed as the standard deviation divided by the absolute mean of each feature. CoV-based stability was classified as excellent, good, or poor for CoV ≤ 5%, 5% < CoV ≤ 10%, and CoV > 10%, respectively. ICC(2,1) assessed repeatability and reproducibility across repeated acquisitions within each insert relative to variability between phantom inserts, with ICC(2,1) > 0.85 classified as good and ≤ 0.85 as poor. Analyses were cross-referenced by insert type and preprocessing condition to identify the most robust configurations, using thresholds from radiomics literature [4], [16], [17].

All 48 preprocessing configurations were applied to 50 phantom scans, and features were extracted for fat, External Water, PVP-40, and FBG tissue (Table 1). Although the phantom includes diffusion-mimicking compartments, no diffusion-weighted imaging was performed; analyses used only SSFP intensity information. Pipeline performance was summarized by the number of features with CoV ≤ 5% within each material group and ICC(2,1) > 0.85 both across all material groups and within each group, following Michalet et al. [4]. Analyses were performed at two temporal levels: intrasession repeatability from five same-day consecutive scans and intersession reproducibility across all 50 scans acquired over 10 days.

## 3 Results

**Fig. 2:**
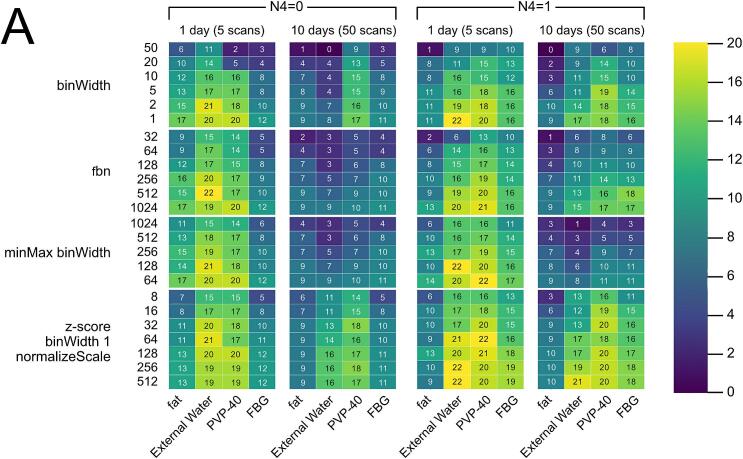

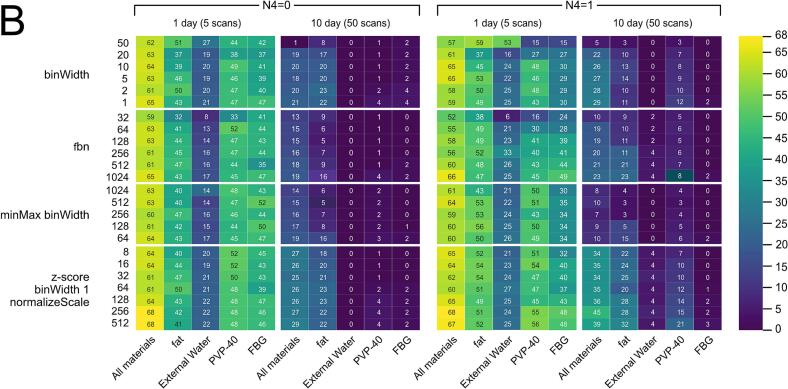

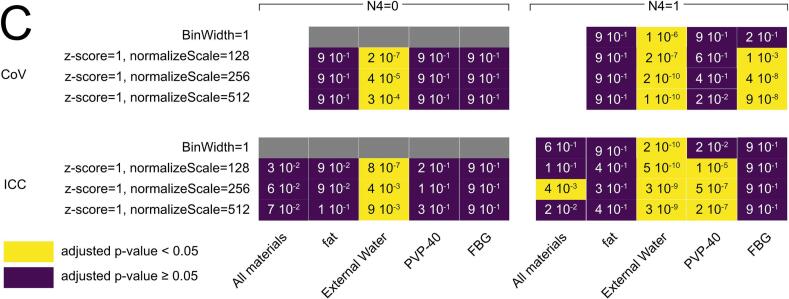
Distribution of stable radiomic features across preprocessing strategies. A: CoV-based evaluation of radiomic feature stability. For each preprocessing strategy, the heatmaps show the number of radiomic features (out of 68) with a coefficient of variation (CoV) ≤ 5%, indicating high stability. Each row corresponds to a specific preprocessing pipeline, and each column represents one of the material inserts (fat, External Water, PVP-40, or FBG). Results are shown for intrasession stability (five consecutive scans acquired within a single day) and intersession stability (50 scans acquired over 10 days). The left panel shows results without N4 bias field correction, while the right panel includes N4 bias field correction. The color scale indicates the number of stable features, ranging from 0 to 20. B: ICC-based evaluation of radiomic feature stability. For each preprocessing strategy, the heatmaps show the number of radiomic features (out of 68) with an intraclass correlation coefficient (ICC(2,1)) ≥ 0.85, indicating high reproducibility. Each row corresponds to a specific preprocessing pipeline, and each column represents one of the material inserts (all materials, fat, external water, PVP-40, or FBG). Results are shown for intrasession stability (five consecutive scans acquired within a single day) and intersession stability (50 scans acquired over 10 days). The left panel shows results without N4 bias field correction, while the right panel includes N4 bias field correction. The color scale indicates the number of stable features, ranging from 0 to 68. C: Evaluation of the improvement in radiomic feature stability with z-score normalization relative to the baseline configuration (BinWidth = 1 without N4 bias field correction). *P*-values were obtained from one-sided Wilcoxon signed-rank tests and adjusted for multiple comparisons using the Benjamini-Hochberg False Discovery Rate. The top panel corresponds to CoV-based analysis, while the bottom panel corresponds to ICC-based analysis. Yellow indicates preprocessing pipelines associated with a statistically significant improvement after FDR correction (adjusted *p*-value <0.05), whereas purple indicates non-significant differences (adjusted p-value ≥0.05).

Fig. 2 summarizes stable-feature distributions across preprocessing pipelines and materials. N4 bias field correction (N4ITK = 1) combined with z-score normalization and NormalizeScale produced the highest number of stable features, with stability maximized at NormalizeScale 128–256 for most materials. Configurations without N4ITK and with low bin numbers performed poorly, often yielding fewer than ten stable features, especially for fat and FBG masks. FBN preprocessing with 128–512 bins performed strongly, especially for PVP-40 and External Water, where more than 20 CoV-stable features were consistently observed (Fig. 2A). Overall, N4ITK correction improved feature stability in most cases.

ICC(2,1) analysis (Fig. 2B) classified individual features by repeatability and reproducibility across the four insert types using all 10 imaging sessions. Feature robustness was quantified as the number of preprocessing pipelines, out of 48, yielding ICC(2,1) > 0.85, both across all materials and within each material group. This analysis complemented CoV by accounting for inter-insert variability. Overall, N4 bias field correction combined with z-score normalization and NormalizeScale 128–512, consistently yielded the most stable features across materials and sessions for both CoV and ICC(2,1).

Intrasession repeatability yielded more stable features than intersession reproducibility across all preprocessing strategies and materials. Pipeline ranking remained consistent between analyses, with N4 correction plus normalization-based methods providing the highest stability. The intra- to intersession decrease was stronger in fat and FBG than in PVP-40 and External Water.

One-sided Wilcoxon signed-rank tests compared each strategy with baseline (BinWidth = 1) using paired CoV and ICC(2,1) feature values, with *p*-values corrected for multiple comparisons using the Benjamini–Hochberg false discovery rate (FDR) procedure (Fig. 2C). For ICC(2,1), z-score normalization significantly improved stability mainly in External Water (*p* = 8 × 10^−7^, 4 × 10^−3^, and 9 × 10^−3^ for NormalizeScale 128, 256, and 512), with a limited effect for the pooled analysis (*p* = 3 × 10^−2^ for NormalizeScale 128). N4 correction independently increased significance for External Water (*p* = 2 × 10^−10^ to 3 × 10^−9^) and PVP-40 (p = 2 × 10^−2^ to 2 × 10^−7^), while fat and FBG remained non-significant. CoV-based Wilcoxon tests showed a similar pattern: z-score normalization improved External Water stability (p = 2 × 10^−7^, 4 × 10^−5^, and 3 × 10^−4^), and N4 correction strengthened improvements for External Water (*p* = 10^−6^ to 10^−10^), FBG tissue (p = 10^−3^ to 10^−8^), and one PVP-40 configuration (p = 2 × 10^−2^).

**Fig. 3:**
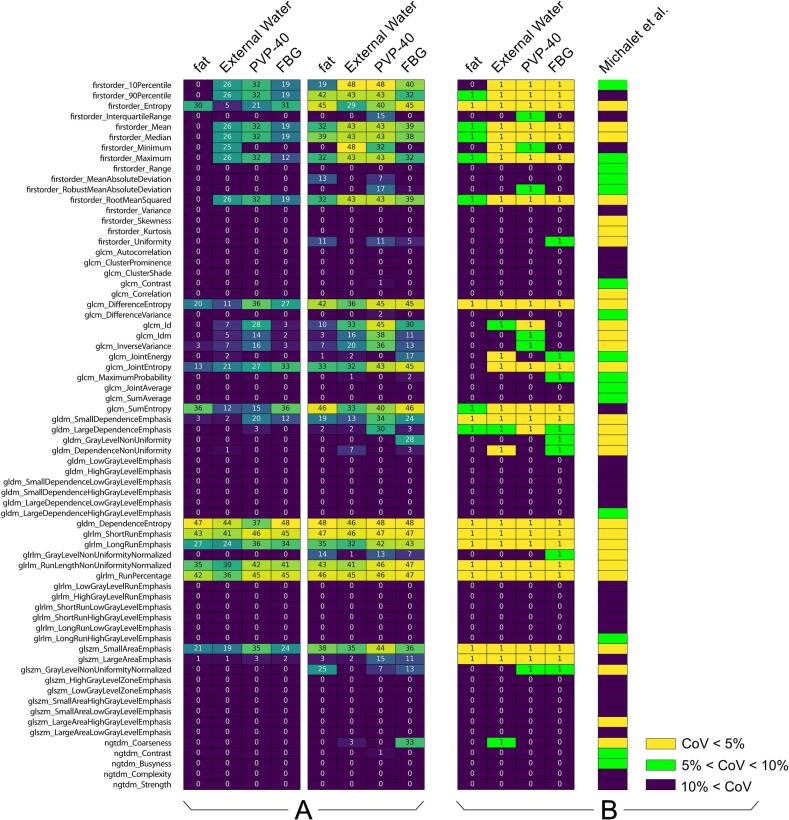
Analysis of individual radiomic features according to their reproducibility. A: For each radiomics feature, the heatmap shows the number of preprocessing strategies (out of 48) for which the feature exhibited a CoV below 5% (left panel) or below 10% (right panel), computed over 10 sessions and across 4 materials (fat, external water, PVP-40, and FBG) B: Corresponding heat map indicating whether the CoV was below 5% (yellow), between 5% and 10% (green), or above 10% (purple) for the preprocessing pipeline using N4 bias correction, z-score normalization, and a bin width of 512 The rightmost column summarizes the reproducibility of the features reported by Michalet et al. [4].

CoV analysis classified individual radiomic features by repeatability and reproducibility across the four insert types using all 10 imaging sessions. For each feature, robustness was quantified as the number of preprocessing pipelines, out of 48, yielding CoV < 5% (Fig. 3A, left) and < 10% (right). This feature-centric evaluation complemented the pipeline-centric analysis and identified material types, such as PVP-40 and External Water, with greater radiomic stability across preprocessing conditions.

Several first-order statistics, including entropy, mean, median, and root mean square, and GLCM/GLRLM features, including Difference Entropy, Joint Entropy, and Run Percentage, showed high stability, with more than 30 pipelines yielding CoV < 5% in stable materials such as PVP-40 and External Water. These inserts consistently produced more stable features than fat and FBG.

By feature class, first-order features were most stable overall. Texture features (GLCM, GLRLM) were more variable: some features, like entropy-based and run-length metrics remained robust, whereas others were more sensitive to preprocessing and tissue heterogeneity. The most robust features are ranked in Supplementary Information by increasing CoV (Table S2) or decreasing ICC(2,1) (Table S3) for each material using z-score normalization (NormalizeScale 512), BinWidth = 1, and N4 bias field correction.

To evaluate generalizability, feature stability was compared with Michalet et al. [4], who assessed radiomic robustness on a 0.35 T MR-Linac using an American College of Radiology (ACR) phantom with repeated phantom imaging and radiomic extraction, providing a benchmark for low-field MRI reproducibility. In our analysis, per-feature robustness across fat, External Water, PVP-40, and FBG was defined by the number of materials with CoV < 5% using the N4 + z-score + NormalizeScale 512 pipeline, then compared with Michalet et al. (Fig. 3B).

## 4 Discussion

This study investigated the reproducibility of MRI-derived radiomic features acquired on a 0.35 T MR-Linac system using a tissue-mimicking phantom with multiple tissue-equivalent inserts. To the best of our knowledge, this was the first MRI-derived quantitative imaging evaluation of a tissue-mimicking phantom in a low-field MR-Linac environment. By incorporating a multi-mask, multi-insert design, this work extended previous single-material phantom analyses and provided material-specific insights into feature stability under different preprocessing pipelines.

These results showed that preprocessing plays a decisive role in determining the reproducibility of radiomic features. Among the 48 tested configurations, normalization-based methods—particularly z-score normalization combined with linear scaling—yielded the highest number of stable features, especially when used with N4 bias field correction, likely by mitigating MRI intensity nonuniformities. These findings align with the consensus in the radiomics literature [18] that bias field correction mitigates low-frequency intensity inhomogeneities inherent to MRI acquisition, thereby improving feature robustness. The Wilcoxon analysis further showed that z-score normalization and N4 correction independently improved stability relative to baseline, with material-dependent effects strongest in homogeneous inserts.

Conversely, low bin numbers and the absence of bias correction led to substantial instability. FBN discretization with 128–512 bins demonstrated strong and consistent performance, confirming previous observations by Duron et al. [19] and Michalet et al. [4] that optimal discretization parameters depend on both the acquisition characteristics and tissue-specific contrast behavior. The sensitivity of radiomic features to preprocessing underscores the need for standardized workflows in MR-Linac-based radiomic studies.

A major contribution of this study was its material-resolved evaluation. The multi-insert phantom design revealed that feature stability depends on the underlying material properties. The PVP-40 and External Water inserts consistently yielded higher reproducibility across preprocessing conditions; in contrast, the fat and FBG compartments showed greater variability.

This material dependence likely reflects differences in tissue-like signal complexity. The PVP-40 and water inserts produced relatively homogeneous MR signals; in contrast, the fat and FBG regions exhibited more complex microstructural and chemical compositions, generating intensity heterogeneity and increased sensitivity to acquisition. This effect may be further accentuated in inter-session analyses, where increased variability introduced by phantom repositioning and session-to-session acquisition conditions can reduce feature stability. This variability mirrors challenges in clinical imaging, where tissue interfaces and heterogeneous textures reduce feature robustness.

In vivo tissues are inherently more heterogeneous than phantom inserts, with complex interfaces, mixed cellular structures, and dynamic physiological processes (e.g., motion, perfusion). Therefore, the variability observed in this study between homogeneous (PVP-40, water) and heterogeneous (fat, fibroglandular) inserts is likely amplified in clinical scenarios, especially affecting texture-based radiomic features sensitive to spatial intensity variations.

These results highlighted the limits of extrapolating outcomes from homogeneous calibration phantoms (e.g. ACR) to realistic anatomical settings. A tissue-mimicking phantom provides a more representative assessment of radiomics performance, bridging controlled phantom conditions and in vivo imaging.

A comparison with Michalet et al. [4], who evaluated radiomic reproducibility using a standard ACR phantom, identified a core set of robust features common to both studies such as glcm_DifferenceEntropy, glcm_JointEntropy, glrlm_ShortRunEmphasis, gldm_DependenceEntropy, and glszm_SmallAreaEmphasis. First-order entropy, mean, median, root mean square, and 90th Percentile also agreed, being stable across multiple insert types and robust in both analyses. Conversely, uniformity, skewness, variance, and maximum probability were unstable or inconsistent across both studies. Our multi-mask, multi-insert design added material-specific insights not offered in Michalet et al. [4], showing higher stability in PVP-40 and External Water than in Fat and FBG. The recurrence of these features across distinct phantom designs and acquisition systems supports their potential use as stable radiomic biomarkers in low-field MRI. This potential has also been reported in longitudinal prostate cancer imaging using a 0.35 T MR-Linac during treatment [20].

However, the present study extended previous work by showing that these features depend on material composition, underscoring the need to account for material- or tissue-specific effects when developing standardized radiomic biomarkers for MR-Linac applications [21].

Despite the methodological advantages of a tissue-mimicking phantom, some limitations remain. First, although the phantom simulated various tissue contrasts, it could not capture the full dynamic variability of in vivo tissues, including motion, perfusion, or temperature-related relaxation effects. Second, the 0.35 T field strength limited the signal-to-noise ratio, potentially increasing discretization-dependent variability.

Future studies could extend this framework to higher-field MRI systems and in vivo acquisitions [22], [23]. Furthermore, harmonization across institutions is needed to establish reference preprocessing pipelines and ensure repeatability and reproducibility of MRI-derived imaging biomarkers in clinical MRgRT workflows, given the influence of imaging conditions [24].

From a clinical perspective, the findings showed that even under controlled phantom conditions, feature stability varied substantially with both material and preprocessing strategies. This has direct implications for MR-Linac-based radiomics, where tissue heterogeneity and image standardization remain major challenges [22]. Robust biomarker development should therefore prioritize preprocessing pipelines that balance normalization, discretization, and bias correction and should validate feature stability across heterogeneous, tissue-like materials rather than relying solely on homogeneous phantoms.

In conclusion, this study demonstrated the feasibility of quantitative radiomic analysis on a 0.35 T MR-Linac using a tissue-mimicking phantom with multiple tissue-equivalent inserts. The multi-mask, multi-insert design enabled material-specific evaluation of feature reproducibility, showing that the PVP-40 and External Water compartments were more stable than the Fat and FBG inserts. These results highlighted the importance of using physiologically relevant phantoms to assess the robustness of MRI-derived radiomic biomarkers and caution against directly extrapolating outcomes from simplified phantoms to clinical imaging.

Future work should focus on validating these reproducible radiomic features in clinical cohorts to assess their potential as prognostic and/or predictive biomarkers of radiotherapy response.

## CRediT authorship contribution statement

**Florian Collard:** Writing – review & editing, Writing – original draft, Software, Methodology, Investigation, Formal analysis. **Fanny Herault:** Software. **Ludovic Vanquin:** Resources, Data curation. **Anne-Laure Gagez:** Supervision, Data curation. **Alain Loussert:** Supervision. **Dominique Collard:** Supervision, Resources, Methodology. **David Pasquier:** Validation, Supervision, Project administration, Methodology, Investigation, Funding acquisition, Conceptualization.

## Funding

This work was funded by Metropole Européenne de Lille (MEL), France.

## Declaration of competing interest

The authors declare that they have no known competing financial interests or personal relationships that could have appeared to influence the work reported in this paper.

## Data Availability

The data supporting the findings of this study are available from the corresponding author upon reasonable request.
