# Supplementary material for: Repeatability and reproducibility of MRI-derived radiomics on a 0.35 T MR-Linac using a tissue-mimicking phantom
Source: Phys Imaging Radiat Oncol. 2026 Jul 7;40:101035. doi: 10.1016/j.phro.2026.101035 (PMC13400326; doi:10.1016/j.phro.2026.101035)
Supplement: Supplementary file 1 — Supplementary material [file mmc1.pdf]

Figure S1

A

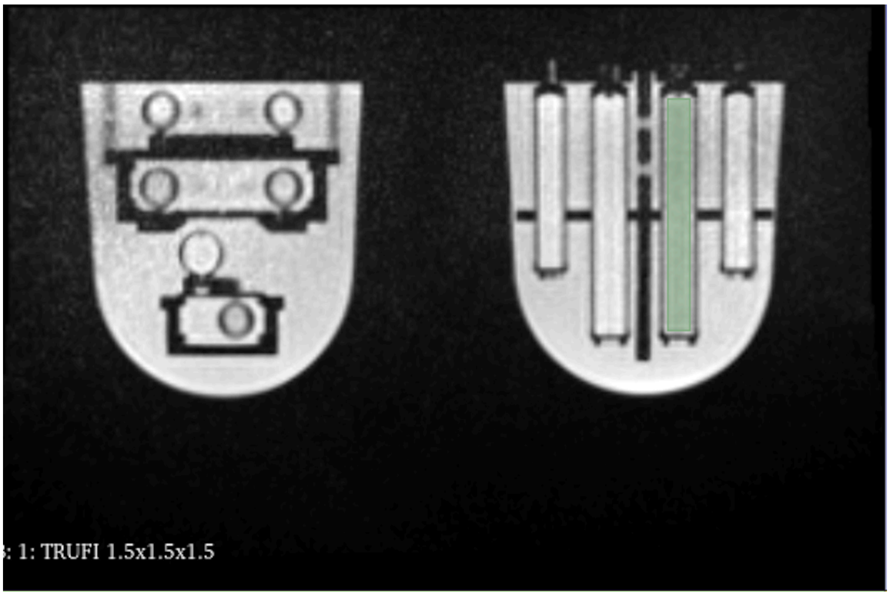

B

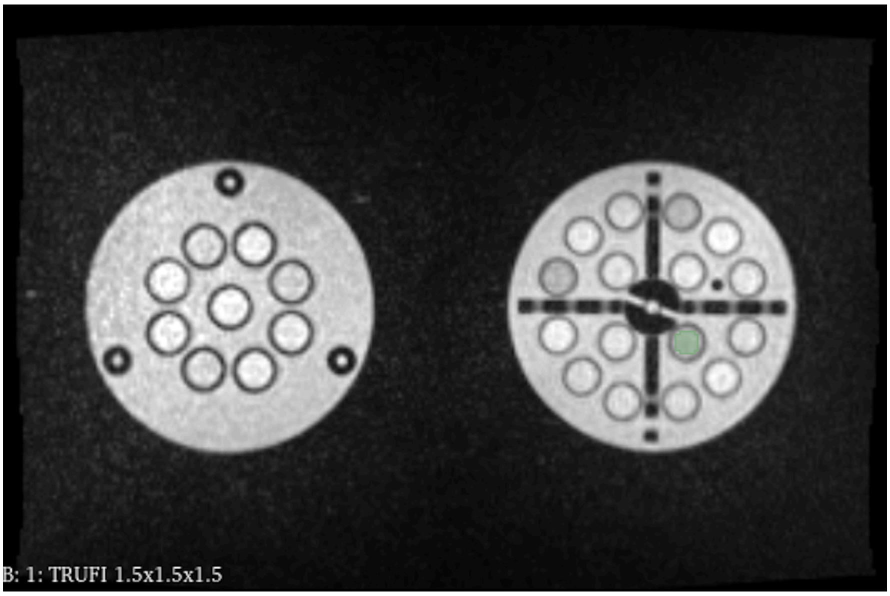

C

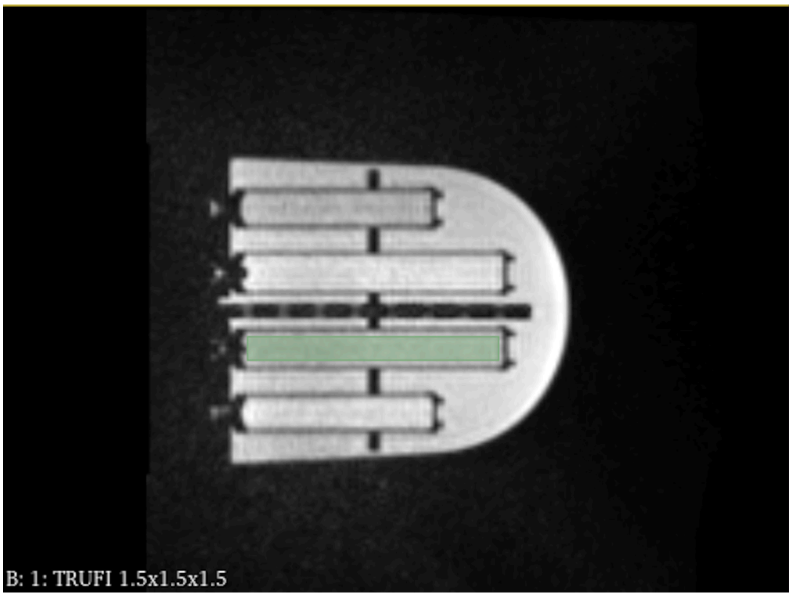

Table S2.A

glrlm_ShortRunEmphasis	0.00060	glcm_Contrast	0.31694
glrlm_RunPercentage	0.00080	ngtdm_Busyness	0.35511
glrlm_RunLengthNonUniformityNormalized	0.00160	glcm_SumAverage	0.39960
glrlm_LongRunEmphasis	0.00241	glcm_JointAverage	0.39960
glszm_SmallAreaEmphasis	0.00786	ngtdm_Coarseness	0.40216
gldm_SmallDependenceEmphasis	0.01559	ngtdm_Strength	0.41861
glszm_LargeAreaEmphasis	0.03145	glcm_MaximumProbability	0.49126
glcm_DifferenceEntropy	0.03277	glszm_LargeAreaLowGrayLevelEmphasis	0.52706
firstorder_Entropy	0.03664	gldm_LargeDependenceLowGrayLevelEmphasis	0.52946
gldm_DependenceEntropy	0.04703	firstorder_Kurtosis	0.53238
gldm_LargeDependenceEmphasis	0.05527	glrlm_LongRunLowGrayLevelEmphasis	0.53261
glcm_SumEntropy	0.06934	gldm_LowGrayLevelEmphasis	0.53318
firstorder_90Percentile	0.07468	glrlm_LowGrayLevelRunEmphasis	0.53318
firstorder_Maximum	0.08409	glrlm_ShortRunLowGrayLevelEmphasis	0.53333
firstorder_RootMeanSquared	0.09046	glszm_LowGrayLevelZoneEmphasis	0.53366
firstorder_Median	0.09097	glszm_SmallAreaLowGrayLevelEmphasis	0.53622
firstorder_Mean	0.09143	gldm_SmallDependenceLowGrayLevelEmphasis	0.53763
firstorder_10Percentile	0.11233	glcm_JointEnergy	0.55239
glcm_JointEntropy	0.12733	ngtdm_Contrast	0.61706
glcm_Id	0.15370	glcm_ClusterProminence	0.64313
firstorder_MeanAbsoluteDeviation	0.15375	gldm_GrayLevelNonUniformity	0.73555
glszm_GrayLevelNonUniformityNormalized	0.16850	glszm_LargeAreaHighGrayLevelEmphasis	0.81546
glrlm_GrayLevelNonUniformityNormalized	0.16934	glszm_HighGrayLevelZoneEmphasis	0.81606
firstorder_Uniformity	0.16944	glrlm_LongRunHighGrayLevelEmphasis	0.81622
firstorder_RobustMeanAbsoluteDeviation	0.17341	glrlm_HighGrayLevelRunEmphasis	0.81637
firstorder_InterquartileRange	0.17646	gldm_HighGrayLevelEmphasis	0.81640
glcm_InverseVariance	0.20835	glrlm_ShortRunHighGrayLevelEmphasis	0.81641
firstorder_Minimum	0.20864	glszm_SmallAreaHighGrayLevelEmphasis	0.81671
glcm_Idm	0.21239	gldm_LargeDependenceHighGrayLevelEmphasis	0.81673
firstorder_Range	0.21450	gldm_SmallDependenceHighGrayLevelEmphasis	0.81781
glcm_Correlation	0.22117	glcm_Autocorrelation	0.86631
ngtdm_Complexity	0.27905	gldm_DependenceNonUniformity	0.86991
firstorder_Variance	0.28874	firstorder_Skewness	2.72481
glcm_DifferenceVariance	0.30511	glcm_ClusterShade	5.05305

Table S2.B

glrlm_ShortRunEmphasis	0.00073	firstorder_RobustMeanAbsoluteDeviation	0.19586
glrlm_RunPercentage	0.00097	gldm_SmallDependenceLowGrayLevelEmphasis	0.19652
glcm_JointEntropy	0.00136	glszm_LowGrayLevelZoneEmphasis	0.19771
glrlm_RunLengthNonUniformityNormalized	0.00194	glszm_SmallAreaLowGrayLevelEmphasis	0.19914
glrlm_LongRunEmphasis	0.00291	glrlm_ShortRunLowGrayLevelEmphasis	0.21280
glszm_SmallAreaEmphasis	0.00887	glrlm_LowGrayLevelRunEmphasis	0.21440
glcm_JointEnergy	0.01334	gldm_LowGrayLevelEmphasis	0.21662
firstorder_Median	0.01778	glrlm_LongRunLowGrayLevelEmphasis	0.22143
gldm_SmallDependenceEmphasis	0.01780	glcm_JointAverage	0.22239
firstorder_RootMeanSquared	0.01918	glcm_SumAverage	0.22239
firstorder_Mean	0.01925	firstorder_Range	0.22267
gldm_DependenceEntropy	0.01967	ngtdm_Contrast	0.23270
firstorder_90Percentile	0.02042	glcm_Correlation	0.24828
glcm_SumEntropy	0.02216	glcm_Contrast	0.27957
glcm_DifferenceEntropy	0.02295	glcm_DifferenceVariance	0.28957
firstorder_10Percentile	0.02496	ngtdm_Busyness	0.29029
firstorder_Entropy	0.02601	glszm_LargeAreaLowGrayLevelEmphasis	0.30226
firstorder_Maximum	0.03413	firstorder_Variance	0.41695
glszm_LargeAreaEmphasis	0.03829	ngtdm_Complexity	0.41760
gldm_DependenceNonUniformity	0.04108	gldm_LargeDependenceHighGrayLevelEmphasis	0.45261
firstorder_Minimum	0.04287	glszm_LargeAreaHighGrayLevelEmphasis	0.45657
gldm_LargeDependenceEmphasis	0.05952	glszm_HighGrayLevelZoneEmphasis	0.47086
glcm_Id	0.09196	glrlm_LongRunHighGrayLevelEmphasis	0.47100
ngtdm_Coarseness	0.09471	glrlm_HighGrayLevelRunEmphasis	0.47220
glcm_MaximumProbability	0.10141	gldm_HighGrayLevelEmphasis	0.47231
firstorder_Kurtosis	0.12326	glrlm_ShortRunHighGrayLevelEmphasis	0.47249
glcm_Idm	0.12558	glszm_SmallAreaHighGrayLevelEmphasis	0.47424
glszm_GrayLevelNonUniformityNormalized	0.12765	ngtdm_Strength	0.47525
glrlm_GrayLevelNonUniformityNormalized	0.13013	gldm_SmallDependenceHighGrayLevelEmphasis	0.47973
gldm_GrayLevelNonUniformity	0.13040	glcm_Autocorrelation	0.49408
firstorder_Uniformity	0.13040	gldm_LargeDependenceLowGrayLevelEmphasis	0.52815
glcm_InverseVariance	0.13120	glcm_ClusterProminence	1.17007
firstorder_MeanAbsoluteDeviation	0.19160	glcm_ClusterShade	4.41839
firstorder_InterquartileRange	0.19569	firstorder_Skewness	4.50703

Table S2.C

glrlm_ShortRunEmphasis	0.00033	ngtdm_Coarseness	0.17700
glrlm_RunPercentage	0.00043	glcm_MaximumProbability	0.18905
glrlm_RunLengthNonUniformityNormalized	0.00087	glcm_JointEnergy	0.23128
glrlm_LongRunEmphasis	0.00130	firstorder_Variance	0.24746
glszm_SmallAreaEmphasis	0.00429	gldm_DependenceNonUniformity	0.25731
gldm_SmallDependenceEmphasis	0.00844	ngtdm_Busyness	0.25855
firstorder_10Percentile	0.01121	ngtdm_Strength	0.27912
firstorder_Mean	0.01170	glcm_JointAverage	0.28004
firstorder_RootMeanSquared	0.01183	glcm_SumAverage	0.28004
firstorder_Median	0.01228	glrlm_ShortRunLowGrayLevelEmphasis	0.28011
glcm_DifferenceEntropy	0.01275	glrlm_LowGrayLevelRunEmphasis	0.28026
firstorder_90Percentile	0.01515	gldm_LowGrayLevelEmphasis	0.28058
glszm_LargeAreaEmphasis	0.01704	glszm_LowGrayLevelZoneEmphasis	0.28084
firstorder_Entropy	0.01888	glrlm_LongRunLowGrayLevelEmphasis	0.28115
gldm_DependenceEntropy	0.02002	gldm_SmallDependenceLowGrayLevelEmphasis	0.28601
glcm_SumEntropy	0.02424	glszm_SmallAreaLowGrayLevelEmphasis	0.28802
firstorder_Maximum	0.02582	firstorder_Kurtosis	0.30285
gldm_LargeDependenceEmphasis	0.02837	glszm_LargeAreaLowGrayLevelEmphasis	0.30420
glcm_JointEntropy	0.03185	ngtdm_Complexity	0.34854
glcm_Id	0.04501	glcm_Correlation	0.40566
glcm_Idm	0.06872	gldm_LargeDependenceLowGrayLevelEmphasis	0.42381
glcm_InverseVariance	0.07339	gldm_SmallDependenceHighGrayLevelEmphasis	0.58295
firstorder_Minimum	0.08081	glszm_SmallAreaHighGrayLevelEmphasis	0.58364
firstorder_InterquartileRange	0.09144	glszm_HighGrayLevelZoneEmphasis	0.58563
firstorder_RobustMeanAbsoluteDeviation	0.09255	glrlm_ShortRunHighGrayLevelEmphasis	0.58665
glszm_GrayLevelNonUniformityNormalized	0.09984	glrlm_HighGrayLevelRunEmphasis	0.58680
glrlm_GrayLevelNonUniformityNormalized	0.10090	gldm_HighGrayLevelEmphasis	0.58689
firstorder_Uniformity	0.10099	glrlm_LongRunHighGrayLevelEmphasis	0.58744
firstorder_MeanAbsoluteDeviation	0.10141	glszm_LargeAreaHighGrayLevelEmphasis	0.59416
ngtdm_Contrast	0.12637	gldm_LargeDependenceHighGrayLevelEmphasis	0.60133
glcm_Contrast	0.13193	glcm_Autocorrelation	0.61941
glcm_DifferenceVariance	0.16496	glcm_ClusterProminence	0.78730
gldm_GrayLevelNonUniformity	0.16551	firstorder_Skewness	2.11753
firstorder_Range	0.17449	glcm_ClusterShade	2.40462

Table S2.D

glrlm_ShortRunEmphasis	0.00057	gldm_SmallDependenceLowGrayLevelEmphasis	0.15703
glrlm_RunPercentage	0.00076	glrlm_ShortRunLowGrayLevelEmphasis	0.15707
glrlm_RunLengthNonUniformityNormalized	0.00152	glrlm_LowGrayLevelRunEmphasis	0.15708
glrlm_LongRunEmphasis	0.00232	glrlm_LongRunLowGrayLevelEmphasis	0.15709
glszm_SmallAreaEmphasis	0.00711	gldm_LowGrayLevelEmphasis	0.15710
glcm_JointEntropy	0.00978	gldm_LargeDependenceLowGrayLevelEmphasis	0.15738
glcm_SumEntropy	0.01211	glcm_Idm	0.16370
gldm_DependenceEntropy	0.01321	ngtdm_Strength	0.17374
firstorder_Entropy	0.01397	glcm_Correlation	0.17677
gldm_SmallDependenceEmphasis	0.01429	firstorder_Minimum	0.17939
glcm_DifferenceEntropy	0.01461	glcm_InverseVariance	0.18011
glszm_LargeAreaEmphasis	0.03091	glcm_Contrast	0.23133
firstorder_10Percentile	0.03467	glcm_DifferenceVariance	0.23741
firstorder_Mean	0.03624	firstorder_Variance	0.24286
firstorder_RootMeanSquared	0.03629	ngtdm_Contrast	0.25517
firstorder_Median	0.03670	firstorder_Range	0.26508
firstorder_90Percentile	0.03837	ngtdm_Complexity	0.29379
firstorder_Maximum	0.04590	ngtdm_Busyness	0.31106
gldm_LargeDependenceEmphasis	0.05530	glcm_JointAverage	0.43335
gldm_GrayLevelNonUniformity	0.05666	glcm_SumAverage	0.43335
glcm_JointEnergy	0.06141	firstorder_Kurtosis	0.51208
gldm_DependenceNonUniformity	0.06831	glcm_ClusterProminence	0.67819
glszm_GrayLevelNonUniformityNormalized	0.07920	glszm_SmallAreaHighGrayLevelEmphasis	0.80031
glrlm_GrayLevelNonUniformityNormalized	0.07972	gldm_SmallDependenceHighGrayLevelEmphasis	0.80085
firstorder_Uniformity	0.07983	glszm_HighGrayLevelZoneEmphasis	0.80091
glcm_MaximumProbability	0.09762	glrlm_ShortRunHighGrayLevelEmphasis	0.80174
glcm_Id	0.10024	glrlm_HighGrayLevelRunEmphasis	0.80177
ngtdm_Coarseness	0.10612	gldm_HighGrayLevelEmphasis	0.80185
firstorder_MeanAbsoluteDeviation	0.10875	glrlm_LongRunHighGrayLevelEmphasis	0.80193
firstorder_RobustMeanAbsoluteDeviation	0.10971	glszm_LargeAreaHighGrayLevelEmphasis	0.80324
firstorder_InterquartileRange	0.11345	gldm_LargeDependenceHighGrayLevelEmphasis	0.80618
glszm_SmallAreaLowGrayLevelEmphasis	0.15676	glcm_Autocorrelation	0.84672
glszm_LowGrayLevelZoneEmphasis	0.15680	firstorder_Skewness	1.38942
glszm_LargeAreaLowGrayLevelEmphasis	0.15694	glcm_ClusterShade	2.62851

Table S3.A

glcm_JointEntropy	0.999
glcm_JointEnergy	0.999
gldm_DependenceNonUniformity	0.999
gldm_GrayLevelNonUniformity	0.985
glcm_MaximumProbability	0.974
glcm_SumEntropy	0.973
firstorder_10Percentile	0.967
firstorder_Median	0.963
firstorder_RootMeanSquared	0.963
firstorder_Mean	0.963
ngtdm_Coarseness	0.961
gldm_SmallDependenceLowGrayLevelEmphasis	0.957
firstorder_90Percentile	0.955
glszm_LowGrayLevelZoneEmphasis	0.955
glszm_SmallAreaLowGrayLevelEmphasis	0.955
glrlm_ShortRunLowGrayLevelEmphasis	0.953
glrlm_LowGrayLevelRunEmphasis	0.953
gldm_LowGrayLevelEmphasis	0.953
glrlm_LongRunLowGrayLevelEmphasis	0.952
gldm_DependenceEntropy	0.949
glcm_DifferenceEntropy	0.948
glcm_Id	0.947
glszm_LargeAreaLowGrayLevelEmphasis	0.929
glcm_Idm	0.927
ngtdm_Contrast	0.922
firstorder_RobustMeanAbsoluteDeviation	0.917
firstorder_MeanAbsoluteDeviation	0.916
glcm_InverseVariance	0.915
firstorder_InterquartileRange	0.914
firstorder_Entropy	0.914
glcm_Contrast	0.907
ngtdm_Strength	0.894
glcm_DifferenceVariance	0.892
firstorder_Maximum	0.889

glszm_GrayLevelNonUniformityNormalized	0.877
glrlm_GrayLevelNonUniformityNormalized	0.877
firstorder_Uniformity	0.877
firstorder_Variance	0.863
gldm_LargeDependenceLowGrayLevelEmphasis	0.852
gldm_SmallDependenceEmphasis	0.830
glrlm_RunPercentage	0.829
glszm_LargeAreaEmphasis	0.828
glrlm_RunLengthNonUniformityNormalized	0.828
glrlm_LongRunEmphasis	0.828
glrlm_ShortRunEmphasis	0.828
glszm_SmallAreaEmphasis	0.825
ngtdm_Complexity	0.821
gldm_LargeDependenceEmphasis	0.821
glcm_Correlation	0.779
firstorder_Range	0.707
ngtdm_Busyness	0.664
firstorder_Minimum	0.645
glcm_ClusterProminence	0.561
glcm_JointAverage	0.558
glcm_SumAverage	0.558
gldm_SmallDependenceHighGrayLevelEmphasis	0.446
glszm_SmallAreaHighGrayLevelEmphasis	0.443
glrlm_ShortRunHighGrayLevelEmphasis	0.440
glszm_HighGrayLevelZoneEmphasis	0.440
glrlm_HighGrayLevelRunEmphasis	0.440
gldm_HighGrayLevelEmphasis	0.440
glrlm_LongRunHighGrayLevelEmphasis	0.439
glszm_LargeAreaHighGrayLevelEmphasis	0.425
glcm_Autocorrelation	0.416
gldm_LargeDependenceHighGrayLevelEmphasis	0.413
firstorder_Skewness	0.202
firstorder_Kurtosis	0.145
glcm_ClusterShade	0.120

Table S3.B

glcm_JointEntropy	0.999
glcm_JointEnergy	0.999
gldm_DependenceNonUniformity	0.999
gldm_SmallDependenceLowGrayLevelEmphasis	0.997
glszm_LowGrayLevelZoneEmphasis	0.997
gldm_GrayLevelNonUniformity	0.997
glrlm_ShortRunLowGrayLevelEmphasis	0.997
glrlm_LowGrayLevelRunEmphasis	0.997
gldm_LowGrayLevelEmphasis	0.997
glszm_SmallAreaLowGrayLevelEmphasis	0.997
glrlm_LongRunLowGrayLevelEmphasis	0.997
glcm_SumEntropy	0.996
glszm_LargeAreaLowGrayLevelEmphasis	0.990
gldm_DependenceEntropy	0.985
firstorder_10Percentile	0.980
glcm_MaximumProbability	0.980
ngtdm_Coarseness	0.974
firstorder_Entropy	0.973
firstorder_Mean	0.971
firstorder_Median	0.971
firstorder_RootMeanSquared	0.971
gldm_LargeDependenceLowGrayLevelEmphasis	0.959
glcm_DifferenceEntropy	0.956
firstorder_90Percentile	0.954
glrlm_GrayLevelNonUniformityNormalized	0.948
firstorder_Uniformity	0.947
glszm_GrayLevelNonUniformityNormalized	0.947
firstorder_Maximum	0.910
ngtdm_Contrast	0.895
ngtdm_Strength	0.888
glcm_Id	0.867
firstorder_RobustMeanAbsoluteDeviation	0.857
firstorder_InterquartileRange	0.851
firstorder_MeanAbsoluteDeviation	0.825

glcm_Contrast	0.774
glcm_Idm	0.751
glcm_InverseVariance	0.704
glcm_DifferenceVariance	0.700
firstorder_Variance	0.659
firstorder_Minimum	0.605
glszm_LargeAreaEmphasis	0.552
glrlm_LongRunEmphasis	0.542
gldm_LargeDependenceEmphasis	0.540
glrlm_RunPercentage	0.540
glrlm_RunLengthNonUniformityNormalized	0.531
glrlm_ShortRunEmphasis	0.530
gldm_SmallDependenceEmphasis	0.530
glszm_SmallAreaEmphasis	0.515
ngtdm_Busyness	0.410
ngtdm_Complexity	0.407
glcm_Correlation	0.357
glcm_ClusterProminence	0.228
glcm_JointAverage	0.068
glcm_SumAverage	0.068
gldm_SmallDependenceHighGrayLevelEmphasis	0.054
firstorder_Skewness	0.052
glszm_SmallAreaHighGrayLevelEmphasis	0.051
glrlm_ShortRunHighGrayLevelEmphasis	0.048
gldm_HighGrayLevelEmphasis	0.048
glrlm_HighGrayLevelRunEmphasis	0.048
glszm_HighGrayLevelZoneEmphasis	0.048
glrlm_LongRunHighGrayLevelEmphasis	0.047
glcm_ClusterShade	0.041
firstorder_Kurtosis	0.037
glcm_Autocorrelation	0.036
glszm_LargeAreaHighGrayLevelEmphasis	0.035
firstorder_Range	0.035
gldm_LargeDependenceHighGrayLevelEmphasis	0.027

Table S3.C

firstorder_Mean	0.890	glcm_ClusterShade	0.266
firstorder_RootMeanSquared	0.887	ngtdm_Complexity	0.265
firstorder_Median	0.885	firstorder_InterquartileRange	0.262
firstorder_10Percentile	0.877	glcm_JointEntropy	0.260
firstorder_Minimum	0.796	glcm_Correlation	0.258
firstorder_90Percentile	0.753	ngtdm_Contrast	0.231
glcm_JointAverage	0.539	firstorder_Maximum	0.204
glcm_SumAverage	0.539	glcm_MaximumProbability	0.182
ngtdm_Busyness	0.537	firstorder_Kurtosis	0.166
gldm_LargeDependenceHighGrayLevelEmphasis	0.520	glcm_DifferenceVariance	0.126
glszm_LargeAreaHighGrayLevelEmphasis	0.513	ngtdm_Coarseness	0.105
glrlm_LongRunHighGrayLevelEmphasis	0.499	glszm_LargeAreaEmphasis	0.096
glcm_Autocorrelation	0.498	glcm_Idm	0.087
gldm_HighGrayLevelEmphasis	0.497	glcm_DifferenceEntropy	0.086
glrlm_HighGrayLevelRunEmphasis	0.497	glrlm_ShortRunEmphasis	0.085
glrlm_ShortRunHighGrayLevelEmphasis	0.497	glrlm_RunLengthNonUniformityNormalized	0.085
glszm_HighGrayLevelZoneEmphasis	0.495	gldm_SmallDependenceEmphasis	0.084
glszm_SmallAreaHighGrayLevelEmphasis	0.490	glrlm_RunPercentage	0.084
gldm_SmallDependenceHighGrayLevelEmphasis	0.488	glrlm_LongRunEmphasis	0.083
firstorder_Range	0.461	glszm_SmallAreaEmphasis	0.079
firstorder_Skewness	0.359	gldm_DependenceNonUniformity	0.078
glcm_SumEntropy	0.346	gldm_LargeDependenceEmphasis	0.077
ngtdm_Strength	0.346	glcm_Contrast	0.068
firstorder_Entropy	0.330	glcm_Id	0.066
glszm_GrayLevelNonUniformityNormalized	0.328	glcm_InverseVariance	0.060
firstorder_MeanAbsoluteDeviation	0.327	gldm_SmallDependenceLowGrayLevelEmphasis	0.006
firstorder_Variance	0.326	glszm_SmallAreaLowGrayLevelEmphasis	0.004
glrlm_GrayLevelNonUniformityNormalized	0.324	glszm_LowGrayLevelZoneEmphasis	-0.002
firstorder_Uniformity	0.324	glrlm_ShortRunLowGrayLevelEmphasis	-0.007
gldm_GrayLevelNonUniformity	0.324	glrlm_LowGrayLevelRunEmphasis	-0.008
gldm_DependenceEntropy	0.323	gldm_LowGrayLevelEmphasis	-0.008
glcm_ClusterProminence	0.285	gldm_LargeDependenceLowGrayLevelEmphasis	-0.009
firstorder_RobustMeanAbsoluteDeviation	0.281	glrlm_LongRunLowGrayLevelEmphasis	-0.009
glcm_JointEnergy	0.267	glszm_LargeAreaLowGrayLevelEmphasis	-0.011

Table S3.D

glcm_JointEntropy	0.999	firstorder_RootMeanSquared	0.408
glcm_JointEnergy	0.999	firstorder_Mean	0.386
gldm_DependenceNonUniformity	0.996	gldm_LargeDependenceHighGrayLevelEmphasis	0.376
ngtdm_Coarseness	0.960	glcm_Autocorrelation	0.374
gldm_GrayLevelNonUniformity	0.940	glszm_LargeAreaHighGrayLevelEmphasis	0.370
glcm_SumEntropy	0.925	glrlm_LongRunHighGrayLevelEmphasis	0.366
gldm_DependenceEntropy	0.896	gldm_HighGrayLevelEmphasis	0.365
glcm_MaximumProbability	0.886	glrlm_HighGrayLevelRunEmphasis	0.365
firstorder_Uniformity	0.863	glrlm_ShortRunHighGrayLevelEmphasis	0.365
glrlm_GrayLevelNonUniformityNormalized	0.863	glszm_HighGrayLevelZoneEmphasis	0.364
glszm_GrayLevelNonUniformityNormalized	0.862	glszm_SmallAreaHighGrayLevelEmphasis	0.363
firstorder_Entropy	0.856	gldm_SmallDependenceHighGrayLevelEmphasis	0.362
glcm_Correlation	0.822	ngtdm_Busyness	0.358
glszm_LowGrayLevelZoneEmphasis	0.806	firstorder_Minimum	0.356
gldm_SmallDependenceLowGrayLevelEmphasis	0.805	ngtdm_Contrast	0.335
glszm_SmallAreaLowGrayLevelEmphasis	0.801	gldm_LargeDependenceLowGrayLevelEmphasis	0.276
glrlm_ShortRunLowGrayLevelEmphasis	0.783	glcm_ClusterShade	0.227
glrlm_LowGrayLevelRunEmphasis	0.781	glcm_DifferenceVariance	0.221
gldm_LowGrayLevelEmphasis	0.777	firstorder_Skewness	0.207
firstorder_RobustMeanAbsoluteDeviation	0.775	glcm_Contrast	0.196
glrlm_LongRunLowGrayLevelEmphasis	0.769	ngtdm_Strength	0.141
firstorder_InterquartileRange	0.759	glcm_InverseVariance	0.111
firstorder_MeanAbsoluteDeviation	0.722	firstorder_10Percentile	0.109
ngtdm_Complexity	0.661	firstorder_Kurtosis	0.104
glszm_LargeAreaLowGrayLevelEmphasis	0.620	glcm_Id	0.104
firstorder_90Percentile	0.592	gldm_LargeDependenceEmphasis	0.062
firstorder_Range	0.589	glrlm_RunLengthNonUniformityNormalized	0.055
firstorder_Variance	0.571	glrlm_RunPercentage	0.054
glcm_DifferenceEntropy	0.568	glrlm_ShortRunEmphasis	0.054
firstorder_Maximum	0.566	glszm_LargeAreaEmphasis	0.054
firstorder_Median	0.465	glrlm_LongRunEmphasis	0.053
glcm_ClusterProminence	0.460	glcm_Idm	0.047
glcm_JointAverage	0.459	gldm_SmallDependenceEmphasis	0.044
glcm_SumAverage	0.459	glszm_SmallAreaEmphasis	0.037

Table S3.E

glcm_JointEntropy	0.999
glcm_JointEnergy	0.999
glcm_SumEntropy	0.919
gldm_DependenceNonUniformity	0.805
gldm_DependenceEntropy	0.744
firstorder_Median	0.713
firstorder_90Percentile	0.708
firstorder_Entropy	0.704
firstorder_RootMeanSquared	0.701
glcm_DifferenceEntropy	0.696
firstorder_Mean	0.694
glszm_GrayLevelNonUniformityNormalized	0.659
glrlm_GrayLevelNonUniformityNormalized	0.654
firstorder_Uniformity	0.653
firstorder_10Percentile	0.644
ngtdm_Complexity	0.614
ngtdm_Busyness	0.612
firstorder_Maximum	0.572
firstorder_Range	0.568
glcm_MaximumProbability	0.562
ngtdm_Coarseness	0.557
firstorder_Variance	0.550
firstorder_MeanAbsoluteDeviation	0.534
glcm_DifferenceVariance	0.522
glcm_Contrast	0.510
glcm_JointAverage	0.496
glcm_SumAverage	0.496
firstorder_RobustMeanAbsoluteDeviation	0.437
ngtdm_Contrast	0.424
ngtdm_Strength	0.413
firstorder_InterquartileRange	0.410
gldm_SmallDependenceHighGrayLevelEmphasis	0.408
glszm_SmallAreaHighGrayLevelEmphasis	0.407
glrlm_ShortRunHighGrayLevelEmphasis	0.406

gldm_HighGrayLevelEmphasis	0.406
glrlm_HighGrayLevelRunEmphasis	0.406
glrlm_LongRunHighGrayLevelEmphasis	0.405
glszm_HighGrayLevelZoneEmphasis	0.405
glszm_LargeAreaHighGrayLevelEmphasis	0.400
glcm_Autocorrelation	0.397
gldm_LargeDependenceHighGrayLevelEmphasis	0.395
glszm_LargeAreaLowGrayLevelEmphasis	0.394
glszm_LowGrayLevelZoneEmphasis	0.392
glszm_SmallAreaLowGrayLevelEmphasis	0.392
gldm_LargeDependenceLowGrayLevelEmphasis	0.384
glrlm_LongRunLowGrayLevelEmphasis	0.382
glrlm_LowGrayLevelRunEmphasis	0.381
glrlm_ShortRunLowGrayLevelEmphasis	0.381
gldm_LowGrayLevelEmphasis	0.381
gldm_SmallDependenceLowGrayLevelEmphasis	0.380
glcm_Id	0.370
firstorder_Skewness	0.328
glcm_ClusterProminence	0.327
firstorder_Minimum	0.299
firstorder_Kurtosis	0.248
glcm_ClusterShade	0.230
glcm_Idm	0.165
glcm_Correlation	0.137
gldm_GrayLevelNonUniformity	0.134
glcm_InverseVariance	0.065
glrlm_RunLengthNonUniformityNormalized	0.062
glrlm_ShortRunEmphasis	0.060
gldm_SmallDependenceEmphasis	0.057
glszm_SmallAreaEmphasis	0.057
glrlm_RunPercentage	0.057
gldm_LargeDependenceEmphasis	0.054
glszm_LargeAreaEmphasis	0.053
glrlm_LongRunEmphasis	0.053
